# Methodological and Ethical Considerations in the Use of Chordate Embryos in Biomedical Research

**DOI:** 10.3390/ijms26062624

**Published:** 2025-03-14

**Authors:** Laura Maria Mendes Campitelli, Karina Pereira Lopes, Isabela Lemos de Lima, Flávia Batista Ferreira, Nayara Delfim Isidoro, Giovana Magalhães Ferreira, Maria Clara Fioravanti Ponce, Milene Caroline de Oliveira Ferreira, Ludmilla Silva Mendes, Pedro Henrique Ribeiro Marcelino, Matheus Morais Neves, Sandra Gabriela Klein, Belchiolina Beatriz Fonseca, Richard Costa Polveiro, Murilo Vieira da Silva

**Affiliations:** 1 Biotechnology in Experimental Models Laboratory—LABME, Federal University of Uberlândia, Uberlândia 38405-330, MG, Brazil; lauramcampitelli@gmail.com (L.M.M.C.); matheusmoraisneves@gmail.com (M.M.N.);; 2 Faculty of Veterinary Medicine, Federal University of Uberlândia, Uberlândia 38410-337, MG, Brazil; 3 Rodent Animal Facilities Complex, Federal University of Uberlândia, Uberlândia 38400-902, MG, Brazil

**Keywords:** animal embryos, biomedical research, 3Rs, animal ethics, alternatives methods, model embryos, genetic, embryonic development

## Abstract

Animal embryos are vital tools in scientific research, providing insights into biological processes and disease mechanisms. This paper explores their historical and contemporary significance, highlighting the shift towards the refinement of in vitro systems as alternatives to animal experimentation. We have conducted a data review of the relevant literature on the use of embryos in research and synthesized the data to highlight the importance of this model for scientific progress and the ethical considerations and regulations surrounding embryo research, emphasizing the importance of minimizing animal suffering while promoting scientific progress through the principles of replacement, reduction, and refinement. Embryos from a wide range of species, including mammals, fish, birds, amphibians, and reptiles, play a crucial experimental role in enabling us to understand factors such as substance toxicity, embryonic development, metabolic pathways, physiological processes, etc., that contribute to the advancement of the biological sciences. To apply this model effectively, it is essential to match the research objectives with the most appropriate methodology, ensuring that the chosen approach is appropriate for the scope of the study.

## 1. Introduction

Animal experimentation uses comparative medicine to infer and analyze genetic and metabolic similarities between the human organism and different animals. Thus, through animal experimentation, it is possible to develop scientific research aimed at understanding various physio-metabolic responses, in addition to promoting an ethical, reliable, and reproducible alternative for new hypotheses about human development [1,2,3].

However, in the development of research involving animals, it is necessary to pay attention to the quality of life of the animals during the pre-experimental, experimental, and post-experimental stages. This is because, in addition to ethics advocating for intrinsic respect for their lives, when an animal is not well-fed and free from distress, pain, disease, and discomfort, its physiological response changes, jeopardizing the quality of the research results [1,3].

When using an animal model, it is also necessary to meet other requirements, such as minimizing the number of animals to the greatest extent possible, refining the study as much as possible to cause the least harm and stress to the animals, and replacing animal testing with alternative methods like in vitro experiments whenever possible [2,4,5].

Thus, despite a certain dependence on the use of animals in a large part of scientific research, their replacement with alternative models is gaining prominence. These methods aim to reduce the use of animals or refine research involving them, as various metabolic or genetic responses can be tested using computer-based (in silico) or laboratory-based (in vitro) models [6,7].

An example of this is skin-sensitization tests, which previously required the use of animals. However, today, highly effective and reliable in vitro models have replaced live subjects. These advancements align with the principles of the 3Rs (replacement, reduction, and refinement) in animal experimentation, promoting more ethical research practices [8].

The adoption of such models not only ensures accurate testing of allergens and irritants but also reduces the need for animal testing, contributing to more sustainable and humane scientific research.

The use of computer models is an alternative that has emerged in recent years, along with the evolution of computer simulation methods [9], which make it possible to predict the interaction of drugs, pharmaceuticals, and substances with the organism [10], as well as the interaction between the substances themselves and organic molecules, allowing the results of these events to be predicted in a biological environment [11].

This methodology allows various biological events to be mimicked by simulating and modeling biomolecules using machine learning, without animals being used in experiments. In silico techniques for such analyses are varied, such as predictive simulations through structure analysis [12] and virtual screening followed by molecular docking through biophysical and mathematical simulation [13,14].

Still, in the in vitro context, the simulation of complex organisms through cells has evolved into the construction of more robust alternative methodologies, such as the 3D cultivation of pluripotent cells [15], allowing for the simulation of tissues and organs through specific differentiation factors and three-dimensional cultivation matrices, permitting the analysis of drug–tissue interaction [16,17].

In the context of the cells used in alternative methodologies, we have those referring to embryos produced by processes such as in vitro fecundation or those produced by natural mating and removed ex vivo. In addition, other cell lines can be used, such as immortalized cells [18], stem cells, and induced pluripotent cells [19]. It is also possible to use cells taken from specific animal tissues, which still need to be used, but with greater utilization of their structures, reducing the quantity of those used [20,21].

This in vitro cultivation methodology has made it possible to develop real synthetic organs, called organoids [22], which can be developed on a wide variety of platforms, such as chips and plates, and can very closely simulate the condition of an organ or biological system [16], making this a method with extreme potential that is still under development [15,22].

In the case of pharmacological tests, the need for animal models has been reduced and refined with the aid of these methods, as it is possible to use various forms of cell cultures as a screening method for potential new drugs [23]. However, in the case of agrochemical or industrial chemical testing, the use of alternative methods remains underexplored [24].

In in vitro studies, technological advances in scientific research have made it possible to study and analyze the influence of substances at different stages of embryogenesis, which would be difficult to conduct with animal models [25,26].

**Figure 1:**
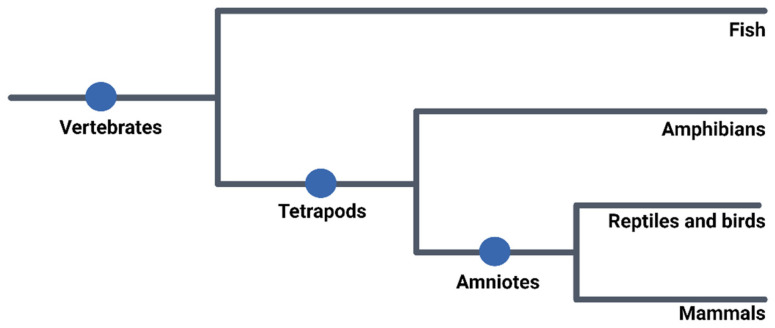
Phylogenetic tree of vertebrate evolution. This phylogenetic tree illustrates the evolutionary relationships among major vertebrate groups, highlighting key branching events. The diagram shows the emergence of tetrapods from ancestral aquatic vertebrates, marking a transition to terrestrial life. The evolution of amniotes further reflects adaptations to dry environments, leading to the divergence of reptiles, birds, and mammals. These evolutionary nodes represent shared ancestry, emphasizing the conservation of embryonic development, physiological processes, and anatomical features. Understanding these relationships provides a framework for comparative research, aiding studies on developmental biology, evolutionary mechanisms, and the use of model organisms in biomedical research.

Embryonic development is the initial stage in the formation of a new individual, beginning with fertilization, a process in which a male gamete unites with a female gamete to form a zygote [27,28]. Following this, the embryo undergoes a series of cleavages, representing a phase of development in which correct physiological functioning is crucial, as any alteration or defect can result in malformations or embryo lethality [29]. This process, with some specific distinctions, is mostly conserved among the chordate groups, given their evolutionary relationship, as shown in Figure 1

Depending on the hypothesis to be tested, a study may use embryos from mammals, fish, amphibians, reptiles, or birds to conduct tests such as teratogenicity, genotoxicity, cytotoxicity, cellular differentiation, gene and protein expression, embryo development and viability, and immunoassays without exposing animals to stressful stimuli or potentially toxic substances [30,31,32].

Thus, given the use of embryos as an alternative model and the need for concise information to select the most appropriate experimentation according to the hypothesis, the present work aims to explore the different embryonic developments and the experimental potential of each in contemporary scientific research.

## 2. Use of Embryos in Research

The use of embryos in scientific research began more clearly around the 1970s for tests involving the analysis of teratogenicity and toxicity of chemical compounds, and over the years, this method has been increasingly studied and refined as a methodology aimed at replacing, reducing, and refining research with laboratory animals [33]. Research using this model generally focuses on observing teratogenicity and embryotoxicity, but it can also explore other aspects, such as the molecular mechanisms involved in cleavage and cell maturation [34,35].

It should be noted that while research aimed at analyzing embryotoxicity seeks to verify toxic effects such as embryonic death, delayed growth, and other types of damage that affect the embryo before it reaches the fetal stage, studies on teratogenic potential specifically look for structural malformations or congenital defects that occur after the embryogenesis period [34].

In general, the main disadvantages of using embryos in research include the inability to analyze the interaction between the mother and the embryo since the tests are performed in vitro or in ovo, limitations in the embryogenesis period that can be studied, difficulty in the detailed observation of certain structures during critical developmental stages, and the fact that it is a technically complex methodology requiring specialized skills and equipment [33,34,36,37].

In addition, there are disadvantages, such as ethical aspects, which prevent the use of some embryonic models [38], as well as morphological variations between the different embryos of the different groups, creating fluctuations in the results which make it difficult to standardize the findings and extrapolate data between species [39]. In addition, some of the methodologies used in these cells do not yet replicate the complexity of the natural development of the animal being studied, thus creating limitations in regard to their use [40].

However, these characteristics do not invalidate the use of this model in preclinical research. There are different embryo models that can be studied, and each has characteristics that make it more suitable depending on the research objective [33].

These problems can also be mitigated by using different approaches, such as standardizing new testing methodologies to help reduce the variability of the embryos chosen [41,42] and using genetically modified embryonic cells to modulate and control metabolic pathways [43].

Research with mammalian embryos mostly aims to understand the effects of chemical substances on embryonic development and reproductive health. Among the species that can be used are large animals, such as cattle and pigs, which are relevant because they have amino acid sequences, chromosomal organization, and physiology more similar to humans than mice, and they are more sensitive to adverse culture conditions than mouse embryos [44]. Additionally, small non-rodent mammals can be studied, as they have a different sensitivity to substances compared to rodents, which may bring their physiological responses closer to those of humans. For example, compounds like thalidomide did not show teratogenic effects in rats and mice but caused morphological alterations in rabbit and human embryos [33]. However, among all possibilities, rodent embryo studies are the most widely used and recommended within this research model [44].

Rodent embryos stand out because they offer practicality, biological relevance, and compatibility with advanced experimental techniques. Their rapid reproductive cycles make it easier to obtain embryos at different developmental stages for experimentation [34]. Additionally, they share a high genetic and biological similarity with humans, making them ideal models for studies on development, genetics, and toxicology [45]. Moreover, many studies accumulated over the years using rodent embryos has created a well-characterized model with a broad reference base, allowing for comparison of results [34,46].

In addition to experiments with mouse embryos, the use of fish embryos, especially zebrafish, is also well-established in chemical safety and toxicology testing of various substances or nanoparticles [47]. The main advantages of this model over others include its rapid life cycle, high reproduction rate, and external fertilization. Zebrafish embryos have differentiated hepatocytes within 36 h of fertilization, allowing for quick screenings of potentially hepatotoxic substances [48].

Additionally, zebrafish embryos are transparent, making it easier to monitor biological processes in real time, such as organ formation and cell migration, without the need for dissection or sample preparation [34,48]. The zebrafish’s genetic features are also well-known, easy to manipulate, and 62% of its genes are orthologous to human genes [48,49].

The use of chicken embryos is recommended for studies aimed at observing angiogenesis and the possible alterations that drugs or toxic substances may cause in this process and in embryonic development as a whole [46]. This model stands out in studies analyzing blood vessels, as they are transparent and easy to visualize in chicken embryos [46].

Additionally, the chorioallantoic membrane (CAM) present in chicken embryos is frequently used in studies on tumor growth and metastasis, as it allows for the implantation of human tumor cells, enabling the observation of tumor growth and behavior [50]. This model also stands out for being simpler and more economical than the rodent embryo model, and the egg provides a controlled physiological environment that is easy to handle and allows for experimental interventions [36,46].

The embryonic development of reptiles involves two main processes: differentiation, which generates tissues and organic systems; and growth, which increases the size of the embryo. Both occur in parallel with the development of extraembryonic membranes [51]. Differentiation begins with neurulation, a reference point in embryological studies, and leads to the formation of membranes, such as the yolk sac and the chorioallantoic membrane, which takes over the gas exchange function [52].

Studies show that embryos respond to temperature variations, suggesting embryonic thermoregulation [53,54,55]. Turtles are often used as models for these studies due to their large eggs, which facilitate the observation of thermal gradients. Transgenesis in reptiles is also gaining prominence, with genetically modified reptiles being used to assess toxic and carcinogenic effects, similar to modified fish and amphibians [56]. Despite these advancements, the use of reptile embryos in research is still less common compared to bird studies.

Lastly, the use of amphibian embryos, especially Xenopus laevis and Xenopus tropicalis, is a classic model in developmental biology studies. These embryos are widely used to study fundamental processes such as gastrulation, germ-layer formation, and organogenesis, and they are also commonly used in studies on cell axis formation and organ development [35,37,57]. The eggs of these animals are large, and their development is rapid, allowing for better manipulation and faster studies. X. laevis embryos reach the blastula stage about 6 h after fertilization, and their eggs can reach 1.5 mm in diameter [35].

Recently, this model has been proposed for studying pigmentation and pigment disorders, as pigmentation changes in Xenopus embryos are easily observable, and their melanocytes respond similarly to those of mammals when exposed to ultraviolet radiation [58]. For toxicity testing, amphibian embryos are recommended for preliminary tests that will need to be complemented by other tests, such as those in rodents [59]. It is evident that the developmental mechanisms in amphibian embryos are highly conserved and applicable to other vertebrates. However, due to their differences from humans, additional studies may be needed when using this model.

**Figure 2:**
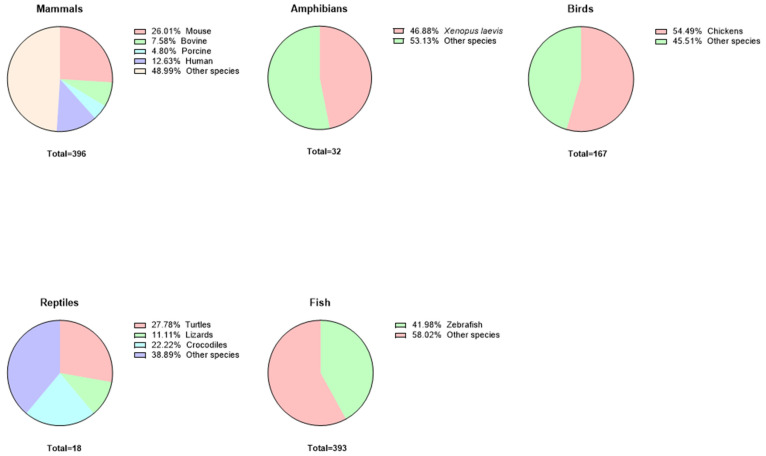
Proportional use of vertebrate embryos in research. These pie charts represent the distribution of research utilizing embryos from various vertebrate groups, with species-specific contributions expressed as percentages of the total studies in each group. In mammals, Mus musculus embryos account for 26.01% of research, while 12.63% involve human embryos, with nearly half (48.99%) using fewer common species. Among amphibians, Xenopus laevis makes up 46.88% of studies, reflecting its prominence as a developmental model. Birds are predominantly represented by chickens (54.49%), while zebrafish embryos contribute 41.98% to fish research, underscoring their utility in genetic and developmental studies. Reptile research is more dispersed, with turtles (27.78%) and crocodiles (22.22%) leading, though “other species” account for a significant portion across all groups. These distributions highlight the variable reliance on model organisms, shaping the landscape of embryology and comparative biology.

Table 1 summarizes information about different types of embryos, which we will discuss in more detail in the following topics. The choice of species to be analyzed as examples in each embryonic group to be discussed in this work was based on an analysis of the literature in the PubMed database, choosing those animals that appeared most often in research in the last 5 years (2019–2024), using the following keywords: (embryo[Title/Abstract]) AND (mammals[Title/Abstract]); (embryo[Title/Abstract]) AND (amphibians[Title/Abstract]); (embryo[Title/Abstract]) AND (reptiles[Title/Abstract]); (embryo[Title/Abstract]) AND (fish[Title/Abstract]); (embryo[Title/Abstract]) AND (birds[Title/Abstract]). The results are graphically expressed in Figure 2.

## 3. Mammalian Embryo

In general, mammalian embryonic development begins with fertilization in the ampulla of the oviduct near the ovary, within the maternal organism [60]. Following this, a totipotent zygote, surrounded by the zona pellucida membrane, will undergo a series of pre-implantation cleavages, leading to the morula and, subsequently, the blastocyst stages [27,28]. This stage is reached after four to six days of development and is characterized by the presence of two distinct cell groups: the inner cell mass, also known as the embryoblast, and the external cells of the trophoblast [61].

**Figure 3:**
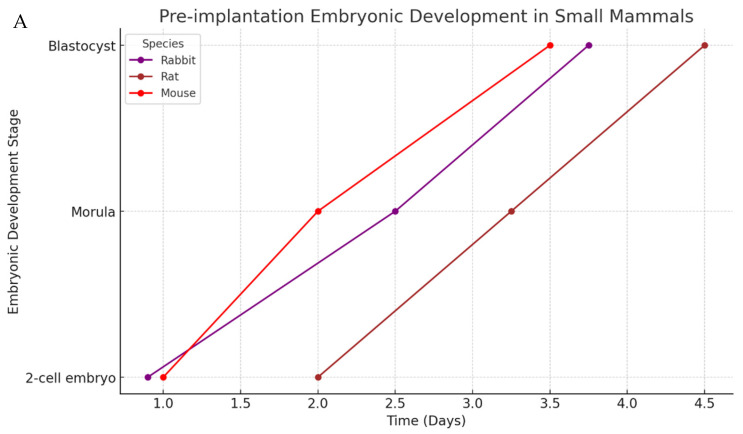

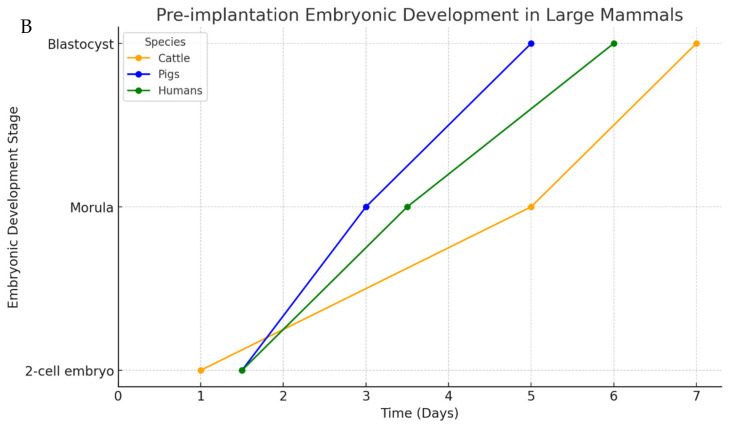
Pre-implantation embryonic development in mammals. This diagram illustrates the key stages of pre-implantation embryonic development in various mammalian species, categorized into small (**A**) (rabbits, rats, and mice) and large (**B**) mammals (cattle, pigs, and humans). The stages depicted range from the initial two-cell embryo to the morula and blastocyst phases. The timeline (in days) provides a comparative view of developmental progression across different species, highlighting variations in the rate and timing of embryonic development. This comparative approach offers insights into the evolutionary adaptations and developmental biology of mammals, contributing to our understanding of embryogenesis and species-specific developmental strategies. These values are averages obtained from the literature. It is important to bear in mind that there are differences among all groups due to species-specific characteristics; however, they remain similar or very close to the values presented.

Following this, the embryo implants in the uterine lining, and once this step is completed, the formation of structures such as the placenta and the yolk sac begins, which are essential for initial growth patterns and the maintenance of fetal development until birth [61]. Figure 3A indicates the pre-implantation embryonic development time for small mammals, while Figure 3B does the same for large mammals

Research on mammalian embryos may have different focuses, but most aim to understand and improve the reproductive health of animals and humans or to investigate the effects and physiological responses of substances on embryonic development. Bovine embryos have a maturation process similar to that of humans, unlike mice, which have a rapid cycle [62].

These animals are of high commercial importance, so research on their embryos focuses on improving assisted reproduction techniques to select genetically desirable traits. They are also used to assess the toxicity of some substances or methods of neutralizing their toxicity [62,63,64,65,66]. Studies on pig embryos do not differ much from those on cattle, but pigs have greater similarity to humans in development, anatomy, and physiology, making them relevant models in studies of human pathologies [44,67,68,69,70,71].

Among small non-rodent animals, rabbit embryos are the most widely used. Like mice, they are easy to handle and produce large litters, but they differ by having a greater resemblance to the human physiological response [33,72]. Research into these animals aims to investigate embryonic development and how different methodologies or toxins may influence it. Thus, this model may be recommended when additional testing is needed to refine and complement results on drug, chemical, and agrochemical testing [72,73,74,75,76].

It is important to note that among the possible models, rodent mammals are still the most used and recommended in embryonic research [33,44]. Despite some discrepancies in development between these animals and humans, such as the stage of zygotic gene activation, which occurs in the two-cell stage in mice and the eight-cell stage in humans [77], comparative medicine principles still allow for the estimation of human embryonic physiological responses by testing on these animals’ embryos [1,3].

Moreover, the ease of manipulating the genome of these animals allows for the creation of phenotypes that mimic a human-like physiological response, potentially reducing existing differences [34]. Rodents are also preferred because they are easy to handle, have fast metabolisms, reproduce quickly, and are raised in highly controlled environments, allowing for pathogen control that could otherwise alter research results [34].

**Figure 4:**
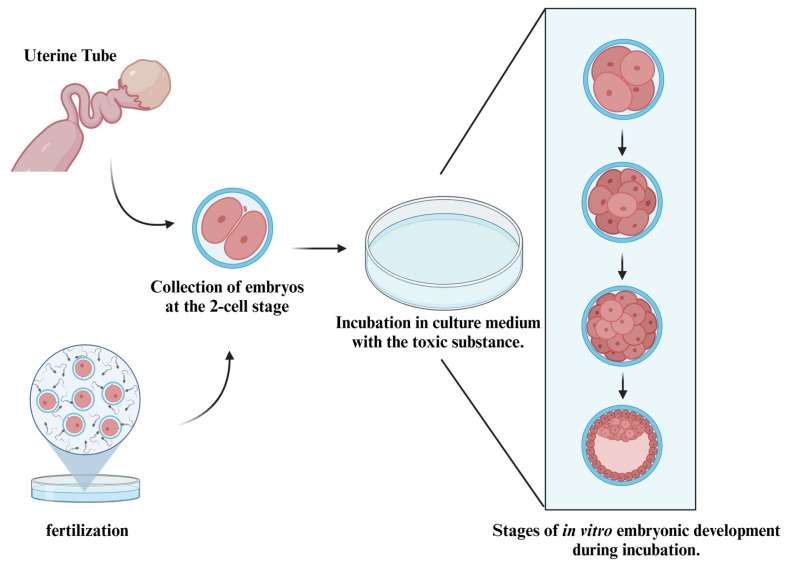
Methodology for in vitro toxicity testing in mammalian embryos. This diagram illustrates the standard methodology used in mammalian toxicity testing, detailing the process from in vitro fertilization to embryonic development under experimental conditions. The procedure begins with the collection of embryos at the two-cell stage, followed by their incubation in a culture medium containing a toxic substance. The diagram further depicts the progressive stages of in vitro embryonic development during incubation, including cell division and differentiation, leading to the blastocyst stage. This approach is widely utilized in toxicology studies to evaluate the impact of various substances on early embryonic development, providing insights into developmental biology and potential teratogenic effects.

Among the possibilities for studies with mammalian embryos, Figure 4 provides a simplified depiction of methodologies utilizing the in vitro exposure method. As explored by Greenlee et al. [78], embryos can be retrieved from the female organism after copulation; however, other protocols may obtain embryos through in vitro fertilization.

Regardless of the method used to obtain embryos, they are collected during the early cleavage stages and incubated in a culture medium mixed with the substance to be tested. In this medium, the embryos can develop up to the blastocyst stage, at which point the study results can be inferred.

## 4. Avian Embryo

**Figure 5:**
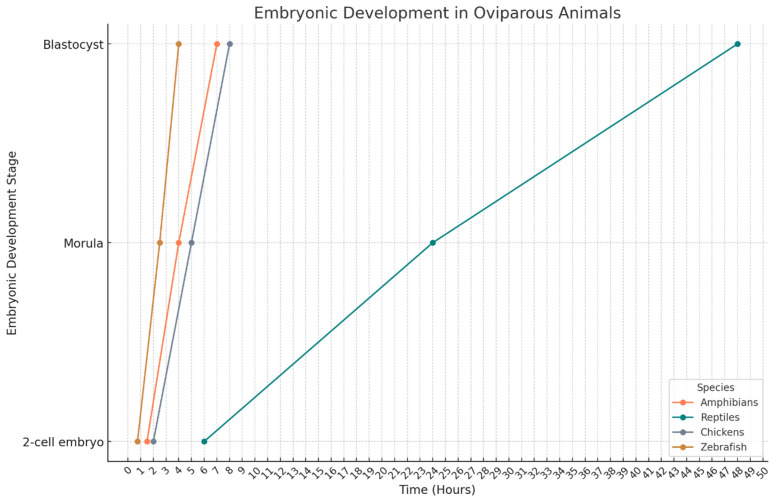
Embryonic development in oviparous animals. This diagram illustrates the key stages of embryonic development in various oviparous species, including amphibians, reptiles, chickens, and zebrafish. The stages depicted range from the initial 2-cell embryo to the morula and blastocyst phases. The timeline (in hours) provides a comparative view of developmental progression across different species, highlighting variations in the rate and timing of embryonic development. This comparative approach offers insights into the evolutionary adaptations and developmental biology of oviparous animals, contributing to our understanding of embryogenesis and species-specific developmental strategies. These values are average and are found in the literature. It is important to bear in mind that there are differences between all the groups, which are due to the specific characteristics of the species but which, however, are similar or very close to the values presented.

The embryonic development of chickens begins with the fertilization of the egg in the female’s oviduct, prior to the secretion of the shell and albumen [60]. Once this step is completed, the first day of development occurs within the animal’s body, during which the embryo undergoes a series of cleavages until it reaches the late blastula stage [60], as described in Figure 5.

After the egg is laid, the embryo continues developing outside the maternal organism, completing gastrulation, neurulation, and histogenesis within 2 to 3 days. By the end of this period, the embryo will also have a circulatory system with a beating heart and a developing nervous system [79,80].

Although there are some morphological differences between the hearts of these animals and that of humans, both possess a four-chambered heart, preventing the mixing of arterial and venous blood [81]. The chicken embryo will then continue its development until hatching on the 21st day [80].

In scientific research, the most used avian embryo model is that of the chicken (*Gallus gallus domesticus*), though there are also references utilizing Japanese quail embryos (*Coturnix coturnix*) [82]. While studies with these embryos do not fully replace classical preclinical research, they are highly relevant as an intermediate step, refining research before testing on mammals [83]. This is possible because, unlike other oviparous species, birds not only share developmental similarities with other chordates but also have a structure homologous to the mammalian placenta called the chorioallantoic membrane (CAM) [83].

Chicken embryos present well-developed vascular tissues that facilitate the study of complex biological systems and offer the possibility of high reproducibility in studies. They are recognized as an intermediate model between in vitro and in vivo research, serving as a preliminary step to mammalian studies, particularly in toxicity studies and drug evaluation, where an evaluation of the organism’s response is necessary, which cannot be replicated in vitro cell culture systems [84].

It is an appropriate model for evaluations, but an obstacle lies in the lack of standardization of its effective use as an experimental model, particularly for drug testing, where there is still no understanding of whether parameters such as quantification of metabolites in the allantoic serum, counting and characterization of blood cells, oxidative stress, and histological alterations are replicable variables from chicken embryo to other in vivo experimental models [85].

Within research, embryos are used for numerous parameters, including angiogenesis, toxicity, ischemia, drug delivery systems, cancer development, and treatment, among other things [86,87,88,89,90]. Emphasis is placed on studies of cancer and metastasis due to the supportive environment for tumors with a large quantity of blood vessels and angiogenesis [91]. They are also widely used as a host system for the replication of various viruses for isolation, for viral titration, and for commercial vaccine production due to possessing cell types that assist in viral replication [92].

Thus, this embryo model provides not only morphophysiological similarities to mammals but also rapid development and ease of manipulation. As a result, these embryos are used from the late blastula stage, after the eggs are laid, through to the end of histogenesis for research on angiogenesis, immune responses, tumor growth and metastasis, substance toxicity, and treatment effects [80,81,82,83,93].

**Figure 6:**
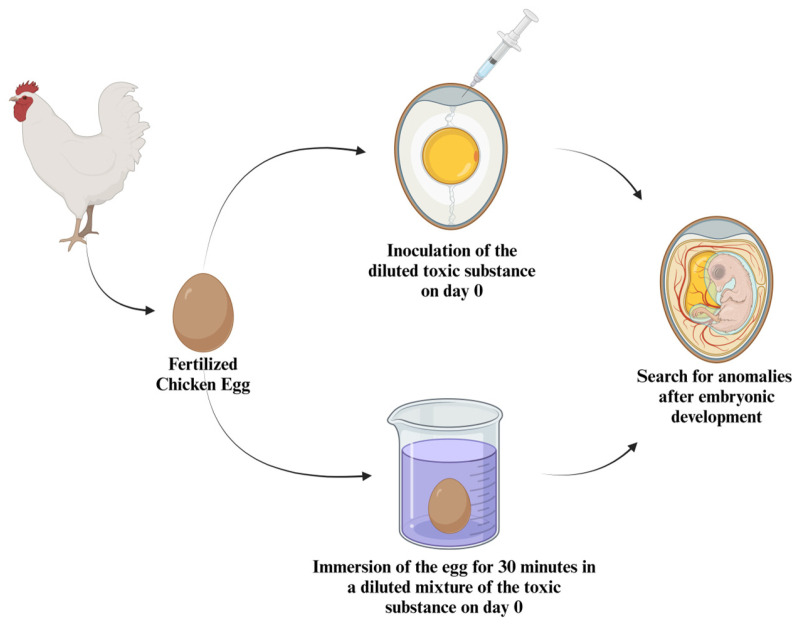
Experimental methodology for toxicity studies using chicken embryos. This schematic represents two methodologies employed in using bird embryos, specifically chicken eggs, as models for studying the toxicity of substances. The process begins with the inoculation of a diluted toxic substance on day 0 for the first methodology or the immersion of the egg in a diluted mixture of toxic substance for 30 min. After embryonic development, the eggs are examined for anomalies. This approach allows researchers to assess the impact of toxic substances on embryonic development, providing valuable insights into developmental toxicity and the potential risks associated with exposure to harmful chemicals.

Based on the methodology described by Lehel et al. [94], it is possible to gain insights into how bird embryos can be used in various ways for toxicological testing. In this study, the toxic potential of two substances was evaluated using chicken embryos, and the adopted methodology explored two distinct exposure methods, as illustrated in Figure 6. It used 40 eggs per group, at a suitable temperature (37–38 °C), relative humidity (65–70%), and daily egg rotation during incubation.

The first exposure method involved the injection of the toxic substance directly into the egg’s air chamber. This approach is more frequently employed in the literature because it allows for a more precise analysis of toxic potential, as the exact amount of substance that comes into contact with the embryo can be controlled.

The second method utilized was the immersion of the egg in a solution containing the toxic substance being tested. This method enables the embryos to be exposed to the substance in a manner more similar to natural environmental conditions, where toxins can penetrate the egg through the shell.

## 5. Amphibian Embryos

One of the most studied animal classes in research due to its ease of study is amphibians [60,95,96]. As anamniotic vertebrates, they lack an amnion surrounding the embryo; however, the development of amphibians employs many of the same processes and genes used by other vertebrates to generate body axes and organs [60].

In this scenario, the importance of using embryos from these animals as models for biomedical research is emphasized, as they enable the understanding of embryogenesis processes that can be applied to the human context.

When discussing the stages of embryonic development, the overall process in amphibians closely mirrors that of other chordates. It begins with fertilization, followed by a series of cleavages that generate the morula, which evolves into the blastula, gastrula, and finally progresses through tissue differentiation [60] at the times described in Figure 5. However, key differences arise during fertilization, which takes place externally. Additionally, amphibian zygotes exhibit a distinctive characteristic—polarity—divided into dorsal and ventral regions. These regions guide the arrangement of both internal and cortical cytoplasm, determining where gastrulation will begin. This process always starts in the ventral portion, although its visibility may vary between species [97].

Among the amphibian species used for this purpose, the one most found in research is the *Xenopus laevis*; the abdomen of an adult female is filled with thousands of large oocytes measuring 1.2 mm in diameter. When removed from the mother, the oocytes can be cultured for several weeks in a simple saline solution [98], thus making them easier to maintain in a laboratory environment and manipulate for research purposes.

Among the advantages of using Xenopus as an experimental system for studying early embryonic development are the ease of animal maintenance, availability of large quantities of embryos throughout the year, and rapid embryo development [95,99]. The embryos are easily manipulated for studies involving tissue transplantation, recombination, and implant cultures [99]. Due to its relatively large embryo size, Xenopus allows efficient isolation of specific regions of embryonic tissues, enabling the supply of sufficient amounts of initial materials for cDNA library construction [99].

Microinjected oocytes have led to many advances in gene expression analysis in vertebrates [98]. After fertilization, a cascade of events begins where the embryo’s cortex rotates relative to the deep region, which will give rise to the dorsal side of the embryo, also known as the “gray crescent” [100]. For many experiments, it is important to predict the location where the dorsoventral axis of the embryo will form; some techniques involving dyes are applied to predict this event [100].

However, during the early stages of developmental genetics research, amphibian embryos were of little use, partly due to the long period of growth of these animals before becoming fertile and because their chromosomes are often found in multiple copies, making the process of mutagenesis difficult [60]. Nevertheless, with the introduction of molecular techniques, such as in situ hybridization, chromatin immunoprecipitation, and dominant-negative proteins, researchers were able to return to studies using amphibian embryos, enabling the integration of molecular analyses with previous experimental findings [60].

Currently, the understanding of embryogenesis is largely associated with the control of gene expression through various signaling pathways. Many of these embryonic signaling pathways that allow embryological events are related to various diseases lacking effective treatments or even their cure [101].

In addition to the previous applications, the embryonic development of the optic pathway of *Xenopus laevis* is one of the most well-understood experimental models for axon localization [102]. This species is also considered to be an established experimental model, with applications for toxicological evaluation of chemical substances and identification of drugs with potential teratogenic risks [95].

The use of amphibian embryos involves various phases of embryonic development, depending on the research objectives. However, some specific phases are more commonly used, including the four-cell embryo phase [95], dorsal marginal zone [103], blastula [96], and gastrulation [95]. In addition, various studies involve the Wnt/β-catenin signaling pathway responsible for embryonic development, with organogenesis, cell differentiation, polarization, and migration [103]. Furthermore, the aquatic nature of embryos, coupled with their independence from maternal influence, facilitates their exposure and absorption of exogenous hormones or other chemical compounds. This enables the study of developmental deficits and functional alterations caused by chemical substances in the embryo [104].

Finally, one of the most important tests using amphibians, especially Xenopus, is the assessment of the toxicity and teratogenic potential of chemical compounds, including various ones used in agriculture, known as the Frog Embryo Teratogenesis Assay (FETAX) [105,106].

**Figure 7:**
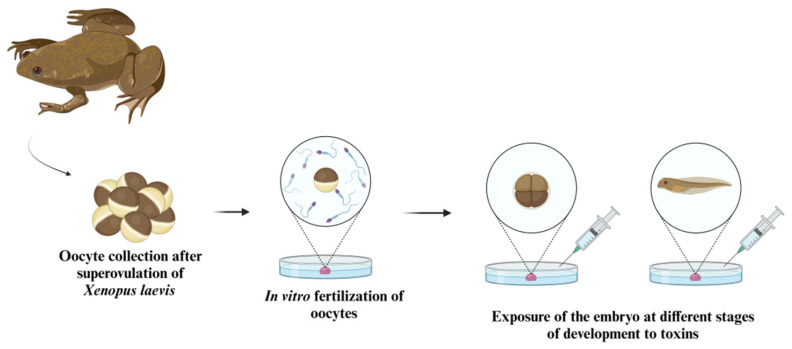
Experimental methodology for toxicity studies using Xenopus laevis embryos. This schematic outlines the common methodology for using amphibian embryos, specifically the Xenopus laevis model, in toxicity studies. The process begins with the collection of oocytes following the superovulation of Xenopus laevis. These oocytes are then subjected to in vitro fertilization. The resulting embryos are exposed to toxins at various stages of development. This method enables researchers to analyze the impact of toxic substances on embryonic growth and differentiation, offering valuable data on the potential risks and effects of environmental toxins on amphibian development.

Among methodologies involving amphibians, Figure 7 provides a simplified representation of how the embryos of these animals can be used in toxicological tests. After fertilization, the embryos can be exposed to the substance being tested by contact with a culture medium mixed with the potential toxin. However, this exposure can occur at different stages of the animal’s development and for varying durations, as discussed in the cited studies.

## 6. Reptile Embryos

The development of reptile embryos involves two distinct processes: differentiation, which is responsible for the origin of tissues and organ systems; and growth, which increases the total size of the embryo. Both differentiation and embryonic growth occur simultaneously with the formation of extraembryonic membranes [51]. The time needed to reach the blastocyst stage—before the formation of the germ layers that will later give rise to complex organs—is illustrated in Figure 5.

Embryonic differentiation begins with neurulation, a phase widely used as a reference in embryological studies due to its frequent occurrence and clear morphological milestones [51]. During neurulation, the mesodermal layer divides to form the extraembryonic coelom, while the definitive yolk sac is established by the vascularized mesoderm-endoderm layer (splanchnopleure) [51]. Despite being the only vascularized membrane in contact with the shell at this early stage, the embryo’s oxygen needs remain relatively low [51].

The allantois appears as the last extraembryonic membrane, developing as an outgrowth of the posterior intestine (involving both the endoderm and mesoderm). Its formation normally coincides with the completion of the amnion and occurs shortly before or concomitantly with the formation of the limbs. The vascularized chorioallantoic membrane (CAM) lining the inside of the shell plays a fundamental role in gas exchange, replacing the yolk sac as the primary respiratory surface in many species [52].

The life-supporting capacity of the CAM makes *Crocodylus niloticus* embryos especially valuable for applied research since their in ovo manipulation can be used to investigate the phasic development of gill arches and odontogenesis [107]. The early stage of development, during the egg window phase, allows for direct interventions in the formation of craniofacial structures, providing answers about dental-regeneration mechanisms that can be applied to therapies in humans, given the similarity of dental anatomy between crocodiles and mammals [107,108].

In the field of comparative research, the accessibility of reptile embryos, which develop externally in many species, allows for in ovo and ex ovo manipulations that provide insights into fundamental developmental processes [109,110]. In reptiles, such as turtles and lizards, the presence of membranes such as the allantois and amnion is crucial for reducing dehydration and protecting the embryo in a dry environment, allowing eggs to survive on land. Turtles have developed eggs with rigid shells, which help prevent water loss, while lizards show variations in the type and thickness of their membranes, which adapt to different habitats and environmental conditions [109,110].

In contrast, birds show even greater complexity in their extraembryonic membranes, with the development of the cocoon, which not only protects the embryo but also facilitates gas exchange, essential for development in varied environments [109,110]. These adaptations exemplify how morphological and functional modifications to the extraembryonic membranes have contributed to the diversification and success of amniotes in terrestrial environments [110].

Reptile embryos have also gained attention as models for genetic manipulation, especially with the advent of CRISPR-Cas9 technologies. Initial attempts at transgenesis in reptiles, such as the work by Modzdiak and Petite (2010) in snakes, culminated in the production of the first transgenic albino lizard using CRISPR-Cas9 [56,111]. These advances have made it possible to generate mutations targeting, for example, the tyrosinase gene, which causes loss-of-function eye defects associated with human albinism, as performed by Rasys et al. (2019) in unfertilized *Anolis sagrei* oocytes [111] by using 21 females with constant crossbreeding, producing large quantities of eggs, all subjected to the methodology created.

In addition, genetically edited embryonic neural stem cells have great potential in restoring the dorsoventral pattern in regenerative tails due to their intrinsic ability to recapitulate embryonic morphogenesis [112,113,114]. These cells are capable of differentiating into specific cell subtypes, such as the dorsal- and ventral-plate domains of the neural tube, as such domains are crucial for the proper formation of the central nervous system and skeletal structures during regeneration [112,115].

**Figure 8:**
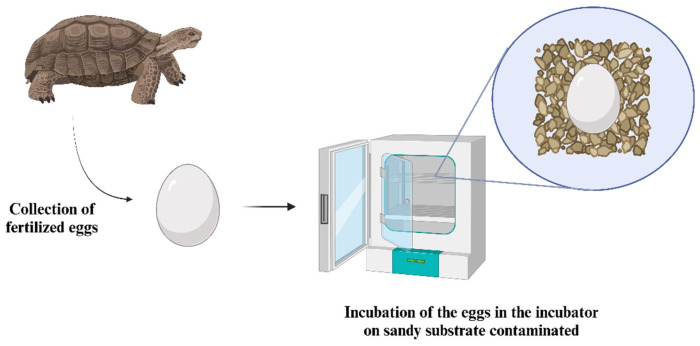
Experimental methodology for toxicity studies using turtle eggs. This schematic depicts the common methodology for employing turtle eggs as a representative reptile model in ex vivo embryonic toxicity studies. The process involves the collection of fertilized eggs, which are then incubated in an incubator or in an environment with a sandy substrate contaminated with the substance under investigation. This setup allows researchers to study the effects of environmental contaminants on the development of reptile embryos, providing insights into the potential impacts of toxins on embryonic growth and survival in reptilian species.

It is also worth emphasizing the importance of such embryos in environmental toxicity research, where monitoring the development of the being inside the egg uses methodologies such as those shown in Figure 8 to exemplify both the potential for aggressive chemical compounds to pass from mother to offspring and to measure the toxicity of the environment, solutions, and products using the egg directly [116,117].

In summary, reptile embryos, by virtue of their unique developmental dynamics and external accessibility, are important and potential experimental models. Not only do they provide insights into fundamental processes such as tissue differentiation, morphogenesis, and regeneration, but they also offer robust platforms for dental, gene-editing, and regeneration studies. As methodologies continue to be refined, the use of reptile embryos in research promises to generate more discoveries that unite developmental biology, evolutionary studies, and biomedical applications.

## 7. Fish Embryos

The embryonic development of fish passes through stages of fertilization, cleavage (which is meroblastic discoidal) [118], blastula, gastrula, segmentation, and subsequent incubation, during which organogenesis occurs. It is noteworthy that in the early stages, the embryo’s DNA is protected from damage by deposits of maternal mRNA [119]. This protection, which has been the subject of preclinical studies, occurs mainly at the blastocyst stage, which is reached in the time described in Figure 5. 

Fish exhibit diverse reproductive strategies influenced by environmental factors. These include hermaphroditism, parthenogenesis, and gonochorism, as well as oviparous and viviparous reproduction [120]. Additionally, there is also the existence of diapause, i.e., dormancy in embryonic development, in some fish populations due to environmental influences [121]. This reproductive plurality allows for the existence of multiple characteristics that make the embryos of these animals promising experimental models for studies ranging from the functioning of genetic mechanisms to ecotoxicity in environments [122,123].

Among the embryos of various animal species, the most prominent ones used as models in research are the zebrafish (*Danio rerio*) and the Japanese medaka (*Oryzias latipes*) [120]. 

In this work, we will focus on the one with the greatest prominence, the zebrafish, as its embryo is the most used model [124]. The first documented use of zebrafish for these research purposes dates back to 2002 [125], although existing research on its development dates back to 1937 [126]. It is noteworthy that the utilization of zebrafish embryos is advantageous as a model for human conditions and related genetic studies, owing to the presence of approximately 70% orthology among genomes, as well as the ease of developing gene overexpression and knockdown processes [127,128].

In the evaluated studies, there is no standardized stage of embryonic development at which the embryos are utilized; they are mostly maintained throughout their evolution in the tests. The treatment timing of the embryos is typically indicated in terms of hours or days post-fertilization [129].

In this context, the primary uses for this model pertain to studies involving toxicity, efficacy, and testing of drugs and substances with potential toxicity. This can be seen in the work performed by Nair et al. in 2021 [130], using groups of 60 embryos produced by crossbreeding in a specific tank at 28 °C, with regular feeding, at a pH of 7.4, creating a detailed protocol on how to harness the study potential of toxic compounds in this model, indicating how to measure the maximum tolerable concentration (MTC), lethal concentration, and compound interaction throughout embryonic and larval development. Such work demonstrates the model’s effectiveness while establishing a standard for its use.

Another study demonstrating this embryo as an effective model for toxic-compound studies involves the detection of cadmium, a heavy metal of medical importance. Developed by Blechinger et al. [125], this study used 25 embryos per group, genetically labeled with fluorescent probes, produced in tanks with healthy adult parents and wild type in a light/dark cycle of 14 h at 28 °C, that respond to different amounts of cadmium in the environment, creating an efficient and reproducible means of indicating the presence and quantity of the metal in the medium.

Molecules with defensive potential against the latter and antioxidant power can also be detected, as shown by Arteaga et al. [131]. Their study demonstrates that zebrafish embryos are models that can replace in vitro antioxidant assays to represent the capacity of molecules in an in vivo context. In this work, nine natural antioxidants in food are used in treatment with a potent oxidant in the presence of embryos, ultimately demonstrating the defense capability of several of these molecules.

The potentialities shown by the above-mentioned works are primarily due to the transparency of this embryo, allowing for the analysis of phenotypic changes caused by such substances, its diminutive size, and the ease of maintaining them in large numbers, owing to their high reproductive capacity and low cost, thus providing a high potential for assay repetition [132].

**Figure 9:**
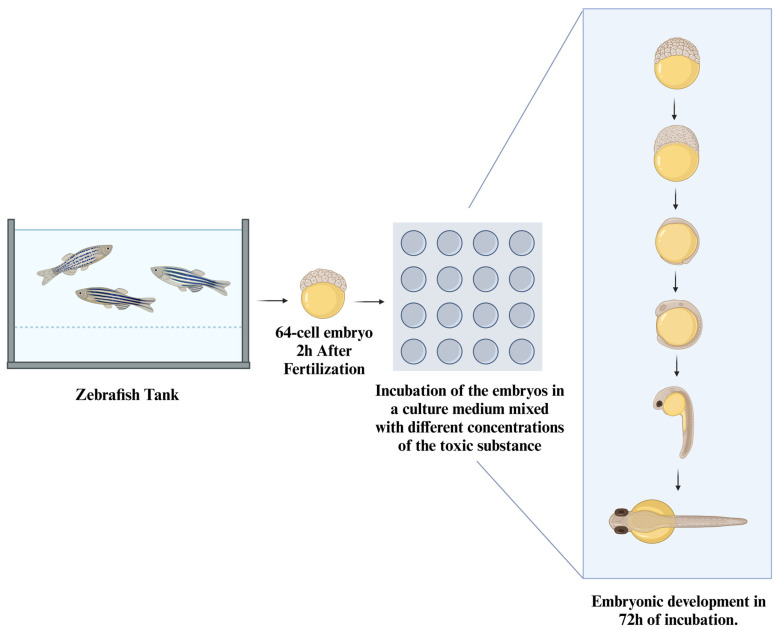
Experimental methodology for toxicity studies using zebrafish embryos. This schematic illustrates the standard methodology for utilizing zebrafish embryos in toxicity testing and other biomedical experiments. The process begins, normally, with the collection of embryos at the 64-cell stage, approximately 2 h post-fertilization. These embryos are then incubated in a culture medium containing varying concentrations of the toxic substance. Over a 72-h incubation period, embryonic development is closely observed. This method allows researchers to evaluate the impact of toxic substances on early developmental stages, offering critical insights into developmental biology and the potential effects of environmental toxins on aquatic organisms. The zebrafish model is particularly valuable due to its transparency and rapid development, making it an excellent system for studying a wide range of biological processes and toxicological responses.

In Figure 9, we have an illustration of how these tests can be conducted. In general, males and females of the species are placed together to mate, and afterward, the eggs containing the embryos can be collected from the tank at different stages of development.

After collecting, the embryos can be placed into multi-well plates containing different mixtures of culture medium and the substance to be tested [133], where their development will be monitored until they reach the larval stage. Subsequently, the results will be analyzed.

## 8. Human Embryo

Embryonic development in humans is highly like that of other mammals, involving internal fertilization and growth. It begins with fertilization, where the sperm interacts with the secondary oocyte, forming the zygote. The zygote undergoes multiple cleavages, giving rise to the morula, followed by the blastocyst, gastrula, and eventually the differentiation and development of tissues [27,28]. The development time for humans is described in Figure 3B.

In biology, a human embryo is something that results from the fusion of the female and male human gametes, which will then go through a series of stages and differentiations [134]. The first of these is known as the pre-implantation period, which takes place in approximately the first 7 days after the fusion, and at this stage, the embryo goes through a series of cleavages until it reaches the blastocyst stage [134].

After this, the embryo begins the post-implantation stage, in which differentiation takes place and, around the 14th day after fusion, the embryo begins the gastrulation process in which the primitive streak is formed, which allows the embryo’s cephalocaudal axis to be recognized, and the three germ layers (ectoderm, mesoderm, and endoderm [135,136,137]) are formed.

At approximately the 8th week of development, the organism will no longer be recognized as an embryo and will be considered a fetus. At this stage, the organs and systems are already initially developed, and the organism will have a human appearance [137].

However, each country can define what an embryo is in its internal legislation [138]. Examples include the Court of Justice of the European Union, which defines “any human ovum after fertilization, any unfertilized human ovum into which the cell nucleus of a mature human cell has been transplanted, and any unfertilized human ovum whose division and further development have been stimulated by parthenogenesis constitutes a ‘human embryo’”; Japan, with the definition, “Embryo—A cell (other than a germ cell) or a group of cells that has the potential to develop into an individual through the process of development in the uterus of a human or an animal and remains at a stage prior to the formation of the placenta” [138]; and the United States of America concluding that “human embryo or embryos’ includes any organism […] that is derived by fertilization, parthenogenesis, cloning or any other means from one or more human gametes or human diploid cells” [138]. This means that different countries have their own regulations about what an embryo is and how studies with human embryo models should be conducted [138].

Thus, while some countries prohibit these studies, others, despite allowing them, have strict regulations that have as a consensus respect for the 14-day rule [139]. This rule has been followed since 1979 and preaches that all experimentation with human embryos cannot extend beyond 14 days after fertilization because, after this period, as the embryo begins the process of gastrulation, it may run the risk of experiencing sensations of pain and suffering [135,140].

Those in favor of relaxing legislation on research into human embryos argue that there are significant differences in anatomy, physiology, and molecular mechanisms between human development and that of other animals, and therefore only the human embryo would provide complete data on its own development [135,139]. However, although each embryonic development model has its own complexity, it is still possible to test different hypotheses in non-human organisms and infer an approximate answer to what it would be like in the human organism [135,141].

In addition to the feasibility of in silico simulation techniques and mathematical and biophysical modeling, it is recommended that, based on the proposed hypothesis, the researcher analyzes the embryonic models available for research and selects the one that will provide the best results, taking into account the need, feasibility, and ethics [23,141]. In Table 1, you can see a comparison of the embryonic development of different species in relation to humans.

However, despite certain temporal and morphological differences, mice and human embryos show similarities in their pre- and post-implantation development, as well as generally showing signaling in their morula stage, which is also present in bovine embryos [142,143]. The zebra fish embryo is similar to a human because it is a chordate animal with a neurocranium balance structures, such as a labyrinth and sensory placoids [23]. The chicken embryo, on the other hand, is amniotic like the human embryo and has interesting physiological structures for tumor studies [135]. Finally, there is also the possibility of studying human embryo models derived from stem cells that would simulate an embryo [144,145,146].

**Figure 10:**
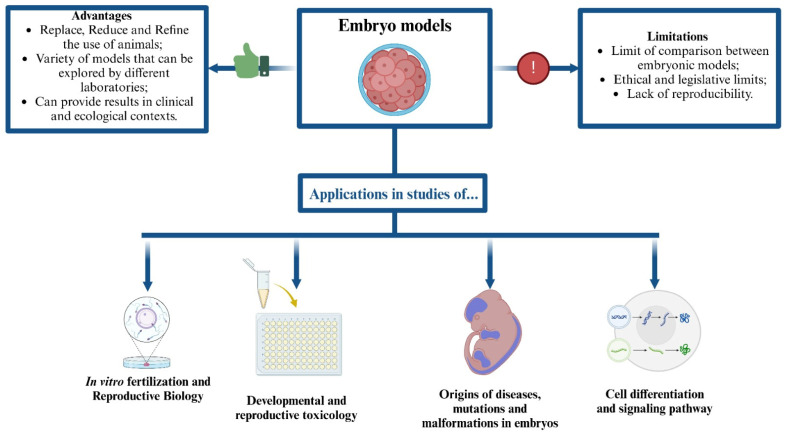
Embryonic models as alternatives in biomedical experimentation. This illustrative figure highlights the use of embryonic models as viable alternatives in biomedical research, showcasing their advantages, limitations, and diverse applications. Key benefits include the potential to replace, reduce, and refine the use of animals in research. These models are employed in studies ranging from vitro fertilization and reproductive biology to developmental and reproductive toxicology, as well as research on the origins of diseases, mutations, malformations, cell differentiation, and signaling pathways. However, limitations such as the restricted comparability between different embryonic models, ethical and legislative constraints, and issues with reproducibility must be considered. By utilizing these models, researchers can achieve meaningful results without relying on human embryos, thereby addressing ethical concerns while advancing scientific knowledge.

Proponents of loosening regulations on human embryo research argue that significant differences in anatomy, physiology, and molecular mechanisms exist between human and other animal development, meaning that only human embryos can provide complete data on human development [135,139]. However, while each embryonic model has its own complexities, it is still possible to test various hypotheses in non-human organisms and infer approximate results for human organisms [135,141], as shown in Figure 10.

## 9. Conclusions

It is noteworthy, finally, the importance of using animal embryos in scientific research, as they serve as important models that can reliably and accurately reproduce tests and metabolic conditions involved in organic processes, which can be extrapolated to various contexts, such as human diseases, toxicity testing of compounds, and observation of genetic events, among other things.

The use of these models is in accordance with all three Rs of animal experimentation, as they allow for a reduction in the use of animals by creating alternative models with a high refinement of results. Moreover, some assays, especially those involving the use of human embryos, are still surrounded by ethical and legal issues in various regions of the world, but they represent an important pathway for the advancement of biomedical sciences.

Due to the genetic and physiological similarities between embryos of different chordate species, it is possible to extrapolate the data found in a model to the human context, when due care is taken. This is possible not only to mimic changes and toxicity to human embryos, but also to simulate an environment for understanding diseases that may affect them.

What is more, due to the functioning of cells with a metabolism very close to that of humans and in vitro culture systems, the use of embryos provides a platform for the study of new drugs and substances to be applied to human health.

It is important to bear in mind that, like all methodologies, there are disadvantages to using chordate embryos in research, especially when it comes to genetic differences, since although they all share a common ancestor, there are marked differences that influence the tests, with mammalian cells generally having a greater genetic similarity for use. When it comes to toxicity research, transparent embryos or embryos that are easier to manipulate, such as those from fish and amphibians, are more suitable than others. In short, there are limitations, but these can be remedied with research that brings the model closer to the target of the study.

We believe that the use of embryos in biomedical research is of utmost importance in replacing in vivo models in studies, ensuring result accuracy and a high reproducibility rate of data. However, we also recognize the need for further studies to address challenges such as the difficulty in extrapolating data from certain models to specific study contexts, the necessity of establishing methodological standards for research utilizing embryos for various purposes, and the need to expand knowledge on the genetic and biochemical characteristics of different types of chordate embryos.

These advancements would allow for a more precise characterization of the model and a better understanding of the changes resulting from experimental assays.

As research progresses, this methodology will enable the development of a reliable platform that significantly reduces the use of animals in substance toxicity testing, the investigation of new molecules and drugs, and the assessment of their biological effects.

## Figures and Tables

**Table 1:** Overview of chordate embryonic models in research. The table highlights key embryonic models, their main traits, applications, advantages, and limitations. Mammalian embryos are crucial for human disease research but costly and ethically regulated. Bird embryos, easy to manipulate, aid cancer and immune studies. Amphibians provide abundant embryos for developmental research, though with longer life cycles. Fish embryos, fast developing and transparent, are ideal for toxicity tests, despite evolutionary distance from humans. Reptiles help explore environmental adaptations but are less commonly used. Human embryos offer unique insights but face significant ethical constraints. These models collectively drive progress in developmental and biomedical sciences.

Embryo Model	Key Characteristics	Main Applications	Main Advantages	Main Disadvantages
Mammals	Development inside the mother; like humans	Reproductive health, toxicity studies, genetic selection, teratogenesis	High genetic similarity to humans; well-characterized	Require strict protocols; higher investment; results inferred without maternal interaction
Birds	External development; has a structure similar to mammal placenta	Cancer research, immune response studies, vaccine studies	Rapid development; easy manipulation	Many tests not standardized
Amphibians	Shares processes and genes with other vertebrates. Amniotic egg; Polarized development	Cell signaling, genetic mapping, toxicity testing, teratogenesis testing	Easy maintenance; produces many embryos	Take time to reach reproductive age
Reptiles	Differentiation and growth vary with environment	Environmental variation studies; toxicity testing	Respond to environmental changes	Less commonly used
Fish	Transparent embryos allow for easy observation	Toxicity testing; safety evaluation	Produce many embryos; low maintenance cost	Greater evolutionary distance from humans
Humans	Use is restricted or limited by the 14-day rule	Embryonic development and disease studies	Unique data on human development	Ethical and legal restrictions in many countries
